# Use of Computer Navigation in Orthopedic Oncology

**DOI:** 10.1007/s40137-014-0047-0

**Published:** 2014-02-22

**Authors:** Kwok-Chuen Wong, Shekhar-Madhukar Kumta

**Affiliations:** 1 Department of Orthopaedics and Traumatology, Prince of Wales Hospital, Shatin, Hong Kong; 2 Department of Orthopaedics and Traumatology, Chinese University of Hong Kong, Prince of Wales Hospital, Shatin, Hong Kong

**Keywords:** Computer-assisted tumor surgery (CATS), Computer navigation, Orthopedic oncology, Sarcoma, Image fusion, Image-to-patient registration, CAD prosthesis, Multiplanar resection, Joint-preserving resection, Tumor patient-specific instrument

## Abstract

The use of computer navigation was first described in the surgical resection of pelvic tumors in 2004. It was developed to improve surgical accuracy with the goal of achieving clear resection margins and better oncologic results. During the past few years, there has been tremendous advancement of computer-assisted tumor surgery (CATS) in the field of orthopedic oncology. Currently, CATS with image fusion offers preoperative three-dimensional surgical planning and allows surgeons to reproduce the intended bone resections in musculoskeletal tumors. The technique is reported to be useful in technically demanding resections, such as in pelvic and sacral tumors; joint-preserving intercalated and multiplanar tumor resection; and complex reconstruction with custom computer-aided design prostheses or allografts. This article provides an up-to-date review of the recent developments and key features in CATS, its current status in clinical practice, and future directions in its development.

## Introduction

Image-based computer navigation has been well utilized in craniomaxillofacial tumor surgery, and studies have demonstrated that the computer navigation technology can improve the precision of various orthopedic surgical procedures, such as spinal pedicle screw insertion, joint arthroplasty, and trauma surgery [1–3]. With the increasing demand for better patient safety and treatment outcomes, it is natural for these well-established principles and techniques to be incorporated in modern orthopedic oncologic surgery. There have been tremendous advancements in computer-assisted tumor surgery (CATS) in recent years, and this article provides an up-to-date review of the recent developments in CATS, its current status in clinical practice, and future directions in its development.

## Overview of CATS in Orthopedic Oncology

Computer navigation surgery allows linking between the patient’s imaging information and anatomy through the use of tracking and registration of the preoperative and/or intraoperative acquired images. This computer-assisted approach has generated great interest among orthopedic oncologic surgeons since Krettek et al. [4] and Hüfner et al. [5] first reported the use of CT-based navigation in pelvic and sacral tumor resection. They used navigation tools to guide the orientation of osteotomies during surgery and concluded that computer-assisted surgery is a potential method to increase accuracy in tumor resections involving anatomic and surgical complexity.

Because CT and MRI are complementary preoperative imaging studies for planning complex musculoskeletal bone tumor resection and reconstruction, Wong et al. [6, 7] later described using CT/magnetic resonance (MR) image fusion in navigation tumor surgery. The two-dimensional (2D)/three-dimensional (3D) fused images enable a detailed delineation of tumor margins, allowing precise definition of the plane of intended bone resection. In a case report, Wong et al. [8] showed the results in a patient with a periacetabular resection and reconstruction with a custom-made pelvic prosthesis using the CATS technique, which incorporated both the planes of intended resection and the location of the pelvic prosthesis at the navigation planning stage. Preoperative navigation planning that includes complete information about resection and reconstruction may be implemented via intraoperative navigation guidance. CATS then may help improve the accuracy of a planned resection and match a reconstruction to a bone resection defect in malignant bone tumor surgery.

The literature regarding basic research in CATS was lacking until Cartiaux et al. [9] described an experimental study in 2008. Under ideal working situations with complete visualization and access to the bone surfaces, four experienced tumor surgeons were asked to operate on simulated plastic pelvic models. The probability of an experienced surgeon obtaining a 10-mm surgical margin with a 5-mm tolerance above or below was only 52 % (95 % CI 37–67). Also, the degree of host-graft contact for reconstruction was found to be poor. This group went on to demonstrate in another experimental study that cutting accuracy was significantly improved with computer navigational guidance [10•]. In a simulated bone model, the error of navigated sawbone cutting was only 2.8 mm, whereas that of the nonnavigated group was 5.2 mm.

More centers began reporting the clinical applications and results of CATS in orthopedic oncology. In a report from Cho et al. [11], three selected femoral tumor cases were operated on under computer navigational guidance with CT-MR fusion images, which maximized the accuracy of bone resection and facilitated joint-preserving resection. The same group (2011) [12•] later described MRI-based navigation as an alternative to CT-based navigation. Direct patient-to-MRI registration could be obtained during surgery by registering the preoperatively placed fiducial markers (absorbable pins). Navigation-guided resection is possible without CT images, and it may avoid exposure to radiation.

In addition to intraoperative guidance of bone tumor resections, Docquier et al. [13] and Gerbers et al. [14•] reported that the same resection planes could be reproduced under navigational guidance to shape the allograft. Better matching of the allograft to the resection defect will improve host-allograft contact, with less chance of nonunion.

Li et al. [15] and Aponte-Tinao et al. [16•] described their early results of multiplanar osteotomies around bone tumors using the CATS technique. Better limb function is achieved after reconstruction if unaffected bone and soft tissue structures are retained.

Computer-aided design (CAD) medical engineering software allows surgical simulation of complex tumor resections and the design of custom prostheses for bone reconstruction. However, this preoperative information cannot be used directly in navigation software because of system incompatibility. Wong et al. [17] developed a method of integrating CAD planning into CATS that enables surgeons to implement any CAD planning with CT-based navigation. This group subsequently reported their use of the new workflow to perform technically demanding joint-preserving tumor resections and reconstructions with custom CAD prostheses in a one-step surgical procedure [18••]. The planned resections not only had clear tumor margins but also had the correct orientation to fit the CAD prostheses.

Larger clinical series with longer follow-up recently were reported. Cho et al. [19••] and Wong and Kumta [20••] further expanded their experience and presented the clinical results of CATS with a minimum follow-up of 3 years. Both groups suggested that CATS may be helpful in safe tumor resection and may lead to a better functional and oncologic outcomes. Jeys et al. [21••] further confirmed this finding in a series of 31 patients with pelvic or sacral malignant tumors undergoing resection with the CATS technique, which reduced intralesional resection from 29 to 8.7 %.

## What are the Key Features of CATS?

**Fig. 1:**
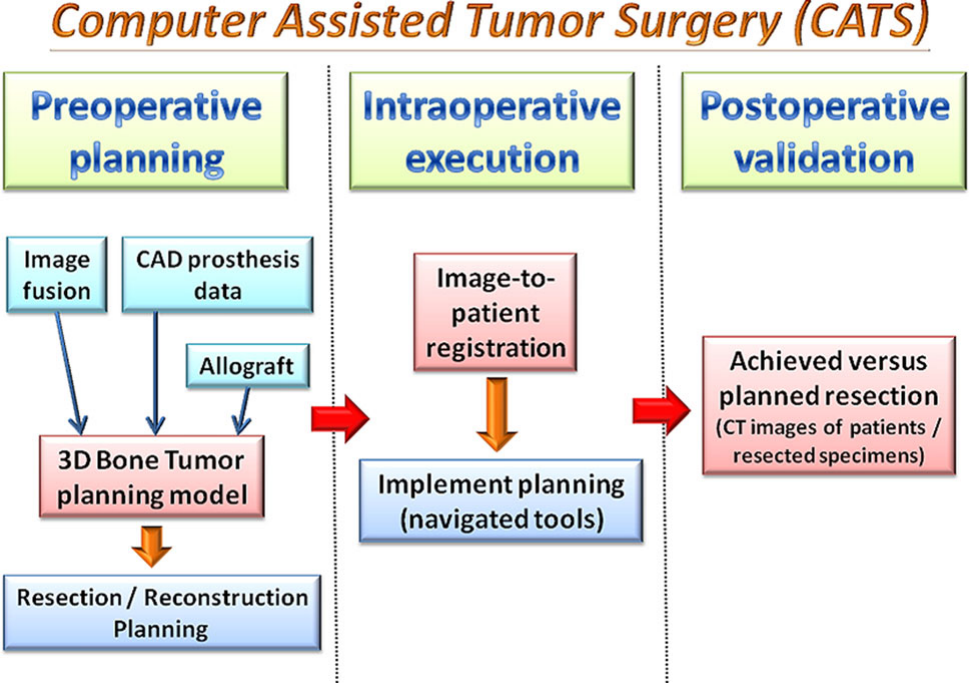
The features and workflow of CATS

In CATS, preoperative computer-assisted planning is as important as the computer navigation used as an intraoperative guiding tool to locate the surgical anatomy. The better and more detailed the planning, the greater the chance the surgical goals will be achieved when the surgeons implement the planning intraoperatively. Some key features are essential to CATS in orthopedic oncology; these features and the workflow of CATS are summarized in Fig. 1. Currently, two commercially available navigation systems (Stryker and Brainlab) can provide most of the key features.

### Preoperative Navigation Planning

#### Multimodal Image Fusion for Resection Planning

**Fig. 2:**
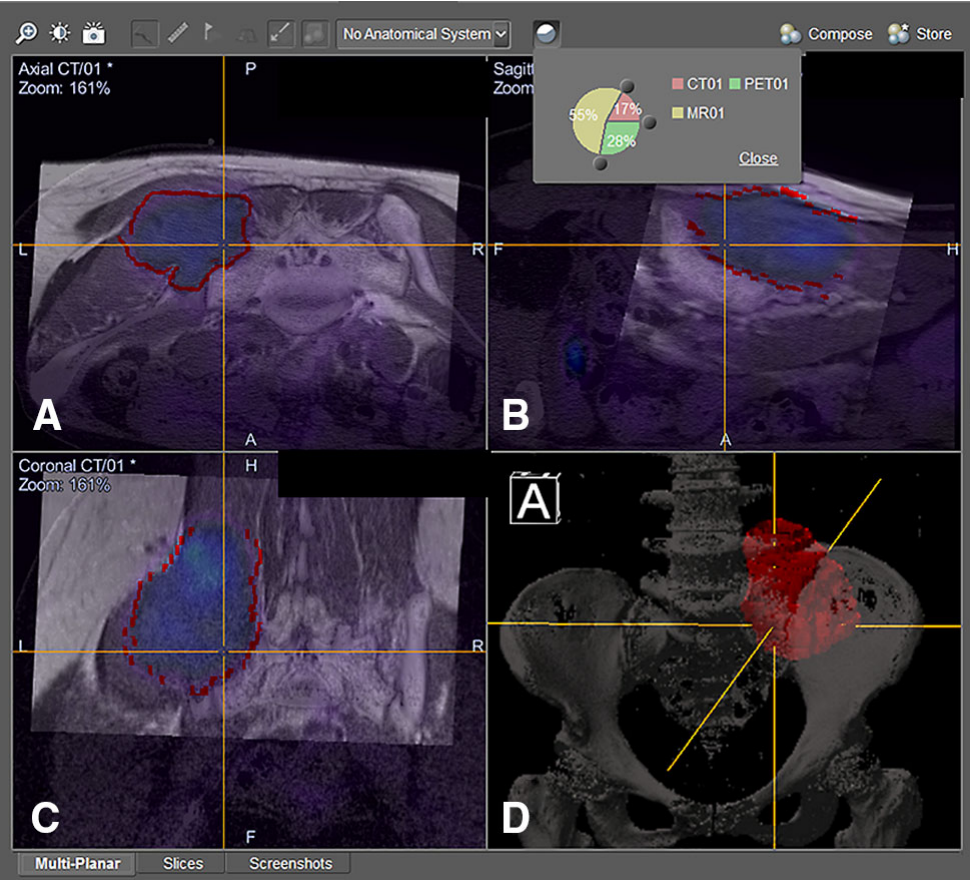
CT/MR/PET fusion images are shown in the navigation display in a patient with a left pelvic tumor involving the posterior superior iliac crest and sacral ala. Wide resection was performed under navigational guidance via a posterior approach, and the left L5 and S1 nerve roots were preserved. Different proportions of image modality could be adjusted on the axial (**a**), reformatted sagittal (**b**), and coronal (**c**) views of the fused images. **d** A 3D bone tumor model was created after the tumor extent was outlined on the MR images. Surgeons then could accurately define the resection planes after studying the fused 2D images and 3D model

CT and MRI are both essential preoperative imaging studies before complex bone tumor surgery [7]. CT shows good bony details, whereas MRI is better at indicating tumor extent and surrounding soft tissue anatomy. Overlaying MRI over CT images with the same spatial coordinates generates fusion images that combine the characteristics of each imaging modality. Tumor extent can be outlined on MRI, whereas a 3D bone model can be created by adjusting the contrast level of the CT images. The MRI-segmented tumor volume and the CT-reconstructed bone model can generate a 3D bone–tumor model. Navigation systems also enable the fusion of functional imaging [positron emission tomography (PET)] scans, which may have the additional benefit of distinguishing tumor from scar tissue in patients with tumor recurrence who had prior resection or radiation. By scrutinizing all the fused image data sets in three spatial dimensions and the 3D model on one screen of navigational display, oncology surgeons can obtain a better mental picture of the tumor’s location and regional anatomy. The best surgical access then can be planned, and vital structures in relation to tumor location can be visualized (Fig. 2). Surgeons can plan and mark the resection planes with specific orientation in one current navigation system (OrthoMap 3D module, version 2.0, Stryker, Mahwah, NJ).

#### Reconstruction Planning

**Fig. 3:**
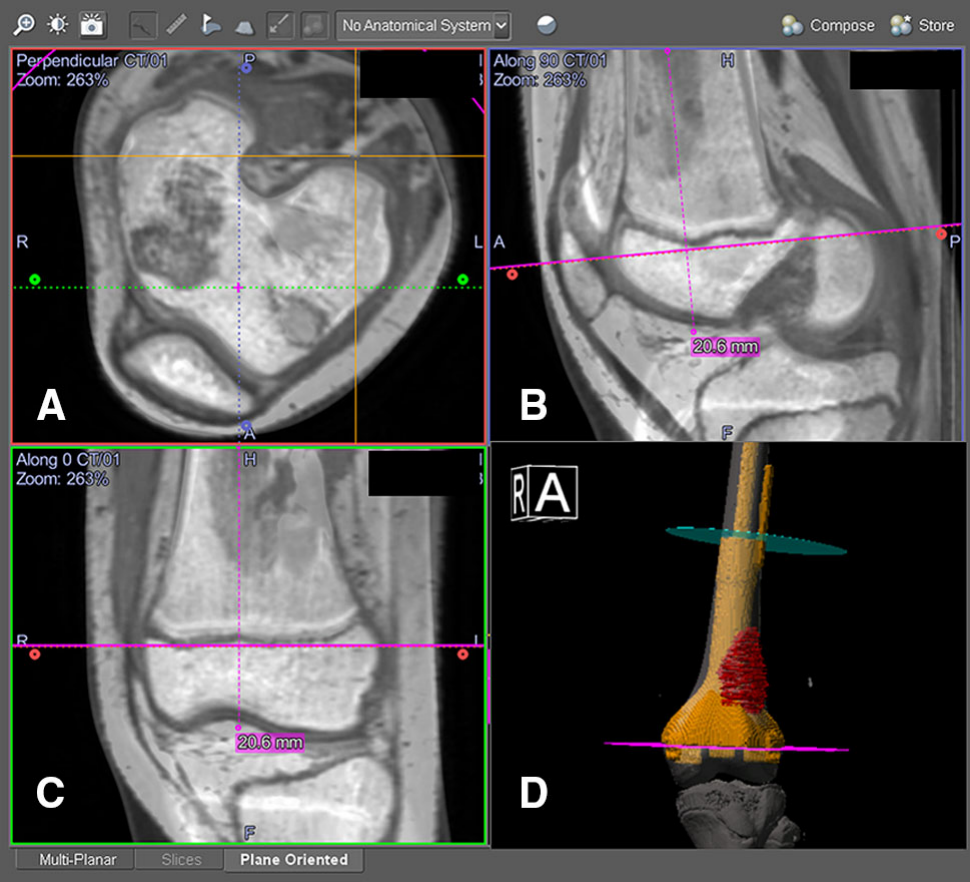
CT/MR fusion images are shown in the navigation display in a 13-year-old girl with a left distal femoral osteosarcoma. Joint-preserving resection and reconstruction were performed with a custom CAD prosthesis. The intraosseous tumor extent was better seen on the T1-weighted MR images. The resection plane (*pink*) was defined as 2 cm proximal to the knee joint. The resection level could be checked with the axial (**a**), reformatted sagittal (**b**), and coronal (**c**) views of the fused images. CAD data of the joint-preserving prosthesis also could be imported into the navigation planning and seen on the 3D model (**d**) with the resection planes. Surgeons checked the final design of the CAD prosthesis before giving approval to the manufacturer

Because the location of the intended resection planes now can be defined precisely in 3D on the preoperative images in the navigation software, planning of the prosthetic or allograft reconstruction after tumor resection is facilitated. Based on the resection navigation planning and preoperative CT images, the implant engineer can design a custom CAD prosthesis to fit the resection defect exactly. Data about the custom prosthesis can be imported into the navigation system. Surgeons can check and modify the design of the custom CAD prosthesis before giving approval to the manufacturer (Fig. 3) [17, 18••].

When a preoperative CT scan also is performed on the allograft with dimensions similar to those of the host bone, the same tumor resection planes may be transferred to the allograft CT data sets in the navigation system. The same resection then may be applied to both tumor resection and allograft shaping under navigational guidance [13, 14•, 16•]. Because the resection planes can be defined clearly on the navigation software, allograft reconstruction is facilitated, and there is a better chance of good allograft-host bone contact.

### Intraoperative Navigation

#### Image-to-Patient Registration

Image-to-patient registration is a process in which the anatomy of the operative site is linked to preoperatively acquired imaging data. It is the most crucial step in CATS during surgery. It is operator dependent and the main determinant of CATS accuracy. The accuracy of the registration may be verified by a navigation probe pointing to specific bony landmarks or running on the bony surface. Unless there is accurate real-time matching between the intraoperative patient anatomy and the preoperative virtual images, we cannot rely on the navigation system to execute the surgical planning and guide the tumor resection.

Registration error, which represents the degree of mismatch between the patient’s anatomy and the virtual preoperative images, is reported in the literature to be <2 mm [15, 19••, 20••, 22•, 23, 24]. Registration errors may vary among different navigation systems, and the amount of registration error acceptable in CATS is not yet defined. A few registration methods are currently used in CATS.

### Manual Registration

Manual registration is the most common method, as the surgical exposure in malignant bone tumor surgery usually provides enough bone surface to perform this registration step. Four to five predefined points on preoperative image data sets are matched with the corresponding points on the patient’s anatomy during the surgery (paired-points matching). The accuracy is improved further by collecting more points from the patient’s bone surface (surface matching). No failure in registration has been reported, and the registration errors at different anatomic sites are reported to be 0.3–0.8 mm in one navigation system (Stryker) [15, 20••, 22•].

### Semiautomatic Registration

So et al. [23] reported the use of CT-fluoro matching for registration. Two fluoroscopic images taken intraoperatively were matched automatically with preoperative CT images after manual image adjustment. The So group found three registration failures with this method of surface matching, and the final resection was more accurate than that of the group with surface matching in its navigation system (Brainlab). However, the amount of registration error by CT-fluoro matching was not mentioned.

### Fiducial Markers

Cho et al. [11] described placing more than three K-wires around bone tumors preoperatively under local anesthesia; CT then was performed. Navigation-assisted procedures were performed after paired-points matching of the locations of the K-wires on CT images. The same group subsequently reported the use of resorbable pins before MRI [12•]. The additional radiation and cost of CT thus may be avoided, and MRI-based navigation then may be performed instead. The registration error was 0.3–1.7 mm with this group’s navigation system (Stryker). Ieguchi et al. [24] reported a less invasive method of using skin markers before CT. However, patients must have CT scanning in the same position as that of the planned surgery, and they should not be obese, as soft tissue deformation during scanning may lead to a discrepancy between the actual position of skin markers and that on CT images. This system (Medtronic) had a registration error of 0.6–1.5 mm.

### Automatic Registration

Intraoperative 3D imaging provides automatic registration on the acquired images. Because the images are acquired intraoperatively, they have the most up-to-date information regarding tumor extent and location. Registration is completed once the imaging is done. It may enable a surgeon to resect small tumors with a minimally invasive approach; however, it has several drawbacks. It requires a radiolucent table for scanning, and patients incur the risk of additional radiation. The image quality is lower than that of a conventional CT scan, and the image volume size (~12 cm^3^) may limit its application in bone tumor surgery.

#### Navigation Tools

Any instrument with a pointed tip and straight axis can be calibrated and tracked by the navigation system. The spatial locations of the tips of the navigation pointer and the surgical instruments, such as diathermy, bone burr, or drill, can be tracked in real time in three dimensions in relation to the patient’s anatomy on the preoperative virtual images. Using the navigation system, locations of planned osteotomies can be identified and marked with diathermy or holes drilled on the bone surface. The resection then may be performed by using an osteotome along the planned orientation guided by the navigation probe. Some navigation systems may support a navigation osteotome so that the depth of the instrument can be better appreciated during resection [21••, 23]. Practically, a navigation saw may also be used for osteotomies. However, ordinary saw blades are too flexible for reliable calibration [14•], and the vibration of the saw blade during an osteotomy may render the navigation instruments inaccurate. Special navigation instruments dedicated to orthopedic oncology should be designed and manufactured to facilitate CATS.

#### Validation of Planned Resection or Resection Margin

Currently, assessing the resection margin during surgery is possible by using frozen sections. A positive result may be used to guide further resection, whereas a negative result adds no information about the distance from the tumor’s edge. Because the dynamic reference trackers are still attached to either the tumor specimen or remaining bone after resection and the image-to-patient registration still remains valid, the achieved resection of the bone ends can be validated intraoperatively under navigational guidance [18••]. By positioning the tip of the navigation probe at the achieved bone resection, surgeons can measure the distance between the virtual tip of the navigation probe and the planned resection on the preoperative virtual images. This not only can further confirm the adequacy of the achieved resection, but also may provide information about the distance from the closest tumor edge.

### Clinical Indications for CATS

For malignant bone tumors, CATS may be beneficial (1) if there are anticipated difficulties in achieving an accurate tumor resection, (2) in obtaining a satisfactory resection plane to accommodate a custom prosthesis, or (3) in shaping an allograft to fit a resection defect [6]. Given the complexity of CATS planning and the additional time required for intraoperative implementation, the CATS technique is better not used for simple resections, but restricted to resections at complex anatomic sites or to more technically demanding resections.

### Pelvic and Sacral Tumors

In the recent cohorts from Cho et al. [19••] and Wong and Kumta [20••], 10 and 12 patients, respectively, had pelvic or sacral tumors resected with the CATS technique. All cases achieved clear resection margins. With a minimum of 3 years of follow-up, the local recurrence rates were 20 % (2 of 10) and 25 % (3 of 12), respectively, which is an improvement compared with 70 % (47 of 67) in a report of 67 pelvic osteosarcomas operated on with traditional surgical techniques [25]. Jeys et al. [21••] recently reported the largest series of 31 cases with pelvic or sacral tumors operated on with the CATS technique. Intralesional resection was reduced from 29 to 8.7 % in this group’s institution, and the local recurrence rate was 13 % at a mean follow-up of 13.1 months [21••]. These three series suggest that CATS may help preserve unaffected sacral nerve roots during sacral tumor resection. The CATS technique appears to be safe, with no specific complications. It improves surgical planning to achieve clear resection margins at complex anatomic sites, such as the pelvis and sacrum, which may offer clinical benefits.

### Joint-Preserving Tumor Resection

**Fig. 4:**
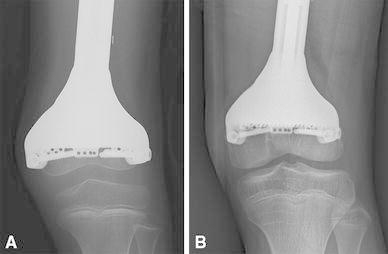
**a** An anteroposterior (AP) radiograph of the left knee shows a joint-preserving tumor resection and custom expandable prosthetic reconstruction in an 8-year-old boy. **b** An AP radiograph of the left knee taken 6 years after surgery shows continuous growth of the remaining distal femur epiphysis. More conservative resection and accurate reconstruction may be performed with the CATS technique, because less soft tissue dissection is required to define the intended resection planes intraoperatively and the blood supply to the remaining epiphysis is preserved

In selected patients, CATS may facilitate precise planning and execution of joint-preserving tumor resections and may enable accurate reconstruction [11, 15, 18••]. Resections that spare native joints and surrounding soft tissues may allow more conservative reconstruction, which may lead to better joint function. Because less bone and its capsular and ligamentous attachments are exposed for reference in marking the resection plane, the blood supply to the epiphysis may be preserved, supporting the continuous growth of the remaining joint in pediatric patients (Fig. 4) [18••].

### Multiplanar Tumor Resection

The CATS technique may facilitate multiplanar osteotomies or hemicortical resections around bone tumors in selected patients [16•, 18••, 20••, 21••]. Compared with uniplanar osteotomies, with these multiplanar osteotomies it is even more difficult to correlate the information obtained from the preoperative images with the real tumor margin at the time of surgery. Because CATS allows accurate planning for complex osteotomies, it may enable surgeons to perform more conservative resections and preserve more unaffected bone for reconstruction.

### Reconstruction with Custom CAD Prosthesis

CATS may allow accurate bone resections that have not only clear margins but also the correct orientation to match the custom CAD prostheses precisely [18••, 20••, 21••]. The accuracy and precision of this one-step operation are difficult to achieve with traditional surgery. CATS has provided a common platform to link surgeons and implant engineers. Implant engineers can design a custom CAD prosthesis with the exact surgical requirements that surgeons define in the navigation software. However, CAD data cannot be imported directly into the navigation system because of system incompatibility. A technique for incorporating CAD data sets into the navigation system has been developed so that CAD custom prostheses can be seen in the navigation system, which greatly helps resection planning and reconstruction [17].

### Limitations

The image seen on the navigation console is a virtual image. Image-to-patient registration is based entirely on bony anatomy and is accurate only as far as bony anatomy is concerned. Following skin incision and surgical manipulation, soft tissues will deform, and the real-time anatomic location will change from that on preoperative virtual images. Only the bony anatomy remains the same. Therefore, CATS enables surgeons to perform accurate bony resection, although soft tissue resection still requires a conventional surgical technique.

Errors may occur during preoperative planning and the intraoperative execution of the CATS technique; these have been discussed in detail [18••]. Surgeons should know and understand these potential errors. Any misinterpretation of the virtual navigational information may result in inaccurate resection and may adversely affect the clinical results. One important error is the time between imaging and surgery. The navigation system is only as good as the raw imaging data; therefore, the time should be short to avoid a discrepancy between the imaging and the patient’s pathology resulting from tumor progression [7].

A method for measuring the accuracy of the CATS technique has not been established firmly. Registration error is only one of the errors that may occur throughout the CATS procedure and should not be regarded as the sole parameter for measuring CATS accuracy [23]. By comparing the discrepancy between the planned and achieved bone resection, CATS accuracy may be better quantified. Various methods have been suggested, including comparison between the planned margin and the histologic margin [23]; the degree of matching of the custom CAD prosthesis to the remaining host bone after resection; the achieved position of the prosthesis obtained from postoperative CT imaging compared with that of the planned [18, 20]; and comparison of the planned resection planes with the ones obtained from CT imaging of the resected specimens [26••]. More investigations are required to determine the standard method and protocol to measure CATS accuracy.

The mean time for navigation preparation or procedures during surgery was reported to range from 24.0 to 28.9 min [19••, 20••, 24]. It has been suggested that although additional operating time was needed for navigation, defining the planned resection plane under navigational guidance actually reduced the overall operating time as surgeons no longer had to guess the resection margins during the surgery [6]. It is anticipated that the additional time will lessen as surgeons become more familiar with the navigation procedures. Extra cost spending on navigation facilities is also a concern as the systems and software are expensive. Studies are required to investigate the cost-effectiveness of navigation procedures with regards to possible improved surgical accuracy in orthopaedic oncology.

Most published papers on CATS are small case series with heterogeneous diagnoses, and there are no reports comparing it with conventional surgical techniques. It remains to be seen whether increasing surgical accuracy actually improves the oncologic and functional results in orthopedic oncology.

### Future Development in CATS

CATS has advanced rapidly in the field of orthopedic oncology, and its workflow is mature. This new technique is feasible, and early, promising results have been reported by different institutions. However, currently, only one commercially available navigation system is dedicated to bone tumor surgery. Although current navigation systems can integrate all the preoperative images for resection planning, they do not support the advanced surgical planning that medical engineering CAD software can provide, such as virtual bone resection and assessment of the resection defect and bone allograft selection from a 3D virtual bone bank [27]. As integrating CAD data into navigation planning becomes possible [17], this advanced CAD planning may be incorporated and then executed with navigational guidance in bone tumor surgery [16•, 18••, 20••]. It will allow more complex resection and reconstruction to be performed accurately in the future. It also will lead to improvement in CAD implant design as surgeons become more capable of performing more complex bone resections with the CATS technique [21••].

With real-time instant visual feedback, intraoperative navigation enables surgeons to locate anatomic and pathologic structures accurately, but the technology involves bulky navigation facilities, a long setup time, and a lack of reliable navigational cutting tools. Tumor patient-specific instruments (PSIs) were recently reported in bone tumor surgery [28•, 29•, 30]. This approach can achieve accuracy similar to that of navigational guidance, but it greatly reduces the operating time and can confine the bone saw along the planned plane of resection. Robotic-assisted tumor surgery (RATS) also was investigated in a sawbone study. It improved the accuracy of wide resection with less deviation from the plan compared with the group using a manual freehand technique [31]. RATS may combine the advantages of both navigation and tumor PSI techniques. More studies are needed to better define its feasibility and clinical results in orthopedic oncology.

With advances in computer technology, an all-in-one computer platform should be developed for customized patient treatment that may allow advanced planning of virtual resection and prosthetic/allograft reconstruction. Surgeons then may choose which tools are most appropriate for their patients.

## Conclusions

In recent years, there have been advances in the development of CATS in the field of orthopedic oncology. CATS with image fusion offers preoperative 3D surgical planning and allows surgeons to reproduce the intended bone resections in bone tumors. The technique is reported to be useful in more technically demanding procedures, such as pelvic and sacral tumor resection, joint-preserving intercalated and multiplanar tumor resection, and complex reconstruction with a custom CAD prosthesis or allograft. However, users must be aware of potential errors in this new technique. Comparative clinical studies with more cases and longer follow-up are needed to determine whether increased accuracy and precision in tumor resection and reconstruction actually may lead to better oncologic and functional outcomes. Future CATS developments may focus on advanced surgical planning with integration between the navigation system and medical engineering software, as well as on the role of tumor PSI and RATS as other tools in bone tumor surgery.
